# HIV-associated neurocognitive disorders at Moi teaching and referral hospital, Eldoret, Kenya

**DOI:** 10.1186/s12883-020-01857-3

**Published:** 2020-07-14

**Authors:** Amina Ali Mohamed, Chrispine Oduor, Daniel Kinyanjui

**Affiliations:** 1 Department of Medicine, Moi University School of Medicine, P.O. Box 4606-30100, Eldoret, Kenya; 2 Department of Mental Health, Moi University School of Medicine, P.O. Box 4606-30100, Eldoret, Kenya

**Keywords:** HIV-associated neurocognitive disorder (HAND), HIV-associated dementia (HAD), Sub-Saharan Africa (SSA)

## Abstract

**Background:**

Human Immunodeficiency Virus (HIV) infection causes a myriad of neurological complications including cognitive deficits referred to as HIV-Associated Neurocognitive Disorders (HAND). With the introduction of combination antiretroviral therapy, there has been an epidemiological shift in cognitive disorders with a decline in the more severe HIV-Associated Dementia (HAD) to an increase in the less severe HAND: Asymptomatic Neurocognitive Impairment (ANI) and HIV-associated Mild Neurocognitive Disorder (MND). Central Nervous System (CNS) involvement in HIV interferes with cognitively demanding activities of daily living and hence a worse quality of life. Early diagnosis is delayed until symptoms are overt.

**Methods:**

We conducted a cross sectional analytical study of HIV infected persons on antiretroviral therapy attending HIV clinic. A systematic random sampling was done to select 360 patients. An interviewer administered structured questionnaire was used to collect socio-demographic data while the CD4 count and viral load were retrieved from the Academic Model Providing Access to Healthcare (AMPATH) database. Pearson’s Chi Square test was used to compare proportions while independent sample t- test was used to compare continuous variables between the patients diagnosed with HAND and those without HAND. Logistic regression model was used to assess the factors associated with HAND.

**Results:**

The mean age of the study participants was 40.2 years. The overall prevalence of HAND was (81.1%) *N* = 292. Mild HAND (ANI and MND) was present (78.6%) *N* = 283, Severe HAND (HAD) (2.5%) *N* = 9. The factors associated with HAND were older age OR: 1.06 (95% CI: 1.03, 1.10), male gender OR: 0.48 (95% CI: 0.24, 0.97), Advanced WHO clinical staging OR: 2.45 (95% CI: 1.20, 5.01) and a higher level of education; secondary/tertiary OR: 0.16 (95% CI: 0.07, 0.38); 0.11 (95% CI: 0.04, 0.35).

**Conclusion:**

The prevalence of HAND in this study population was found to be high (81.1%). Older age and advanced WHO clinical staging were associated with an increased risk of hand while higher level of education and male gender were protective.

## Background

HIV virus has a direct effect on the cellular immune system through depletion of infected CD4 lymphocytes and also has broad effects on the nervous system, including evidence for direct pathology in the brain, spinal cord, and peripheral nerves [1]. Neurological involvement of HIV remains an important problem since antiretroviral therapy (ART) has not fully accomplished full protection of the nervous system.

HIV-Associated Neurocognitive Disorders (HAND) are neurological disorders associated with HIV infection and AIDS. They have a highly variable clinical course and a spectrum of signs and symptoms, ranging from subtle cognitive and motor impairments to profound dementia [2].

A consensus research definition of HIV-associated neurocognitive disorder includes the sub classifications of: Asymptomatic Neurocognitive Impairment (ANI), HIV-associated Mild Neurocognitive Disorder (MND), and HIV-Associated Dementia (HAD) [3].

HAND even in its mild form is associated with less ability to perform the most complex daily tasks, worse quality of life, difficulty obtaining employment, and shorter survival [4]. In the study of individuals with longstanding aviremia, an overall prevalence of cognitive complaints was found to be 27%. The prevalence of HAND was 84% among patients with cognitive complaints and 64% in those without. ANI was present in 24%, MND in 52%, and HAD in 8% [5].

HAND confers an increased risk for early mortality, independent of medical predictors [6, 7] and often interferes significantly with cognitively demanding activities of daily living such as employment, medication management and driving [8–10].

The gold standard for assessment of HAND is a detailed battery of neuropsychological tests, however; they are seldom available to patients in busy settings [3, 11]. Various tools have been developed to help assess the neurocognitive dysfunction. Given the scarcity of the neuropsychological battery of tests, the screening tools such as the Montreal cognitive assessment (MoCA) and the International HIV dementia scale (IHDS) have been utilized in several studies in Sub Saharan Africa (SSA) including Kenya. These include the Montreal cognitive assessment (MoCA), which is more sensitive to the milder forms of HAND (ANI/MND) and the International HIV dementia scale (IHDS), which is more sensitive to the severe form of HAND (HAD). The Lawton Instrumental activity of daily living (IADL) has been used to assess the functional status of the patients, which are mostly impaired in patients diagnosed with HAND.

Despite HAND being an important cause of morbidity and mortality among people with HIV, its prevalence and the associated factors have not been well characterized in our set up.

A modified version of IHDS, the HIV Dementia Diagnostic Test, (by adding neurological and functional status items) was evaluated by Kwasa et al. in Kenya [12]. The modified tool exhibited moderate sensitivity and specificity of 63 and 67% respectively. A study aimed to define the performance characteristics of the IHDS in three East African countries (Kenya, Uganda, Tanzania), using the customary IHDS cut-off of 10, found a sensitivity of 91% with a specificity of 17% [13]. Sacktor et al. validated IHDS in Uganda with a cut off of < 10 for detection of HIV Dementia [14]. The sensitivity was 80% and specificity was 55%. Kiswahili version of the MoCA (K-MoCA, has been recently validated in Tanzania [15] yielding a sensitivity of 70% and a specificity of 60% for Mild Cognitive Impairment, and sensitivity of 72% and specificity of 60% for dementia.

The three East African states have close socio-cultural practices and speak a common Swahili language. Therefore, the tools were used in the study, as the results of the tool validation may not differ significantly.

## Methods

### Study aim, design and setting

A cross-sectional analytical study conducted at Moi Teaching and Referral Hospital (MTRH) to determine the prevalence and the factors associated with HAND.

Adult HIV infected patients on ART were enrolled into care in the AMPATH (Academic Model Providing access to Healthcare) clinic at MTRH. AMPATH currently has more than 80,000 patients on ART. MTRH serves as the teaching hospital for Moi University School of Medicine and is the second largest tertiary referral centre in Kenya. It serves a population of 16 million people (40% of Kenya’s population) in western Kenya and is the primary care site for the 300,000 urban populations in Eldoret town.

**Fig. 1:**
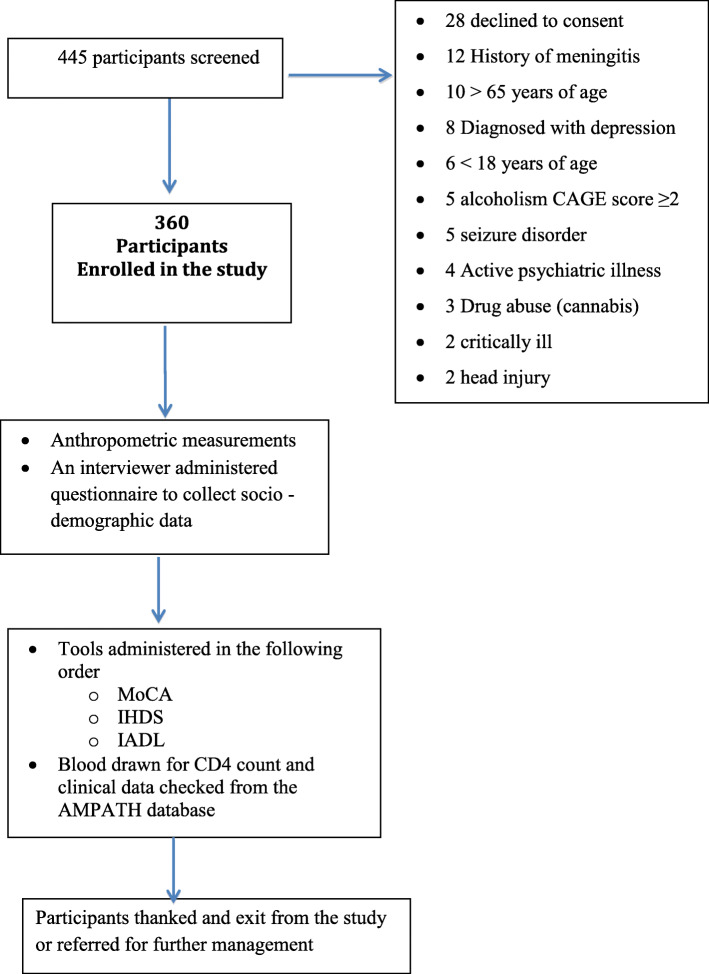
Recruitment schema and study procedure

Inclusion criteria included being HIV infected on ART and age between 18 years and 65 years. The exclusion criteria included active or known CNS opportunistic infection, fever of > 38 °C, history of chronic neurological disorder such as stroke, epilepsy and traumatic brain injury, active psychiatric disorder (presence of delusions, hallucinations and disorganized speech, catatonic behavior during the study evaluation and history of psychiatric disorder actively scrutinized in the medical records and the study questionnaire - Additional file 1), Alcoholism (CAGE score > 2) and drug abuse, severe medical illness that would interfere with the ability to perform the study evaluation (Fig. 1).

### Sample size calculation and sampling technique

Sample size of 360 patients was derived from fishers’ exact formula.

The Sample size is calculated as shown below:

$$ \mathrm{N}=\frac{{\left(\mathrm{Z}\upalpha /2\right)}^2\mathrm{x}\;\mathrm{p}\left(1\hbox{-} \mathrm{p}\right)}{{\mathrm{d}}^2} $$

Where:

N = minimum sample size required

α = the level of significance (5%)

Zα/2 = the value of Z at the selected level of significance

p = likely prevalence (31%) - Ugandan study- N.Sacktor

d = *P* value (0.05)

$$ \mathrm{N}=\frac{\left(1{.96}^2\right)\ast 0.31\ast 0.69}{(0.05)^2} $$

N = 328 patients

10% adjusted for non-response and missing data = 360 patients

Systematic random sampling technique was used to sample the participants meeting the inclusion criteria.

### Data collection, variables and measurement

Participants had their anthropometric measurements: height and body weight measured. They were then taken through the interviewer administered structured questionnaire (Additional file 1) then the tools starting with the MoCA, IHDS then the IADL were administered by a well trained Psychologist who graduated with a Bachelor’s degree from Moi University administered the screening tools. Subsequently, they were sent for the CD4 count at the AMPATH reference lab and the baseline CD4 count and viral load collected from the Academic Model Providing Access to Healthcare (AMPATH) database (Fig. 1).

The primary outcome variables definition: Asymptomatic neurocognitive impairment (ANI) – Based on low scores < 26 in MoCA (Montreal cognitive assessment tool) and good performance in IHDS (International HIV dementia scale tool) > 10 and IADL score of 8.

Mild neurocognitive disorder (MND) – Based on low scores < 26 in MoCA (Montreal cognitive assessment tool) and good performance in IHDS (International HIV dementia scale tool) > 10 and IADL score of < 8.

HIV- Associated Dementia (HAD) - Based on low scores < 26 in MoCA (Montreal cognitive assessment tool) and poor performance in IHDS (International HIV dementia scale tool) < 10 and IADL score of < 8.

The data variables collected included: age, gender, Body mass Index (BMI), level of income, level of education, WHO clinical staging, CD4 count, viral load, duration of HIV (Length of time since diagnosis), duration of HAART, type of ARV’S, Central penetration effectiveness (CPE) score and co-morbidities.

### Statistical analysis

Data analysis was done using STATA. Descriptive statistics for measures of central tendency such as the mean and median were used to summarize continuous variables. The mean and the corresponding standard deviation were used to summarize continuous variables that assumed the Gaussian distribution. Such variables include age and years of education among others. Variables such as CD4, body mass index (BMI), duration of living with HIV, and duration of using HAART among others violated the Gaussian assumptions hence were summarized using the median and the corresponding inter quartile range (IQR). Gaussian assumptions were assessed using Shapiro-Wilk test and histograms. Frequencies and the corresponding percentages were used to summarize categorical variables such as gender, marital status, and education level, WHO clinical stage among others.

Pearson’s Chi Square test was used to compare proportions between the participants diagnosed with HAND and those without HAND while Independent samples t-test was used to compare continuous variables between the participants diagnosed with HAND and those without HAND.

Logistic regression model was used to assess the determinants of HAND. Odds ratios (OR) and the corresponding 95% confidence intervals (95% CI) were reported.

## Results

### Socio-demographic and clinical characteristics

A total of 360 participants with mean age 40.2 (SD: 11.5) years, Range: 18.0–65.0 years were included in the study (Table 1).

**Table 1:** Socio-demographic characteristics

**Variable**	**N**	**Mean (SD) or n (%)**
Age (Years)	360	40.2 (11.5)
Range (Min. – Max.)		18.0–65.0
Male	360	126 (35.0%)
Marital status		
Single		89 (24.9%)
Married	358	161 (45.0%)
Divorced/Separated		38 (10.6%)
Widowed		70 (19.6%)
Education level		
Primary		159 (44.2%)
Secondary	360	155 (43.1%)
Tertiary		46 (12.8%)
Years of education	360	9.9 (3.1)
Range (Min. – Max.)		2.0–18.0
Occupation		
Employed		82 (22.8%)
Self employed	359	89 (24.8%)
Unemployed		188 (52.4%)
Level of income (Kenya Shillings/Month)		
< 10000		302 (86.5%)
10000–50000	349	41 (11.8%)
50000–100000		5 (1.4%)
> 100000		1 (0.3%)

*SD* Standard Deviation

The median BMI was 22.9 (IQR: 20.3, 25.5) kg/m^2^ with 29.2% who were overweight or obese. Twenty-eight (7.8%) of the participants had comorbidities. Hypertension was the predominant comorbidity affecting 6.3% of the total participants (Table 2).

**Table 2:** Clinical characteristics

**Variable**	**N**	**Median (IQR) or n (%)**
Body Mass Index (Kg/m^2^)	360	22.9 (20.3, 25.5)
Range (Min. – Max.)		14.8–54.0
< 18.5		34 (9.4%)
18.5–25.0	360	221 (61.4%)
25.0–30.0		86 (23.9%)
> 30.0		19 (5.3%)
Have comorbidities	360	28 (7.8%)
Comorbidities		
Asthma		1 (3.6%)
Diabetes mellitus		1 (3.6%)
Hypertension	28	23 (82.1%)
Hypertension / Diabetes mellitus		2 (7.1%)
Rheumatic Heart disease		1 (3.6%)

*IQR* Inter Quartile Range

The median baseline and current CD4 were 243.0 (10.8.0, 399.0) cells per mm^3^ and 491.0 (336.5, 701.0) cells per mm^3^ respectively. Fifty (16.4%), and 48.3% had at least 500 cells per mm^3^ at baseline CD4 and current CD4 respectively. Three hundred and three (84.6%) of the participants had suppressed viral load and 160 (46.7%) were in WHO clinical stages 3 or 4 (Table 3).

**Table 3:** HIV treatment and markers of immunity

**Variable**	**N**	**Median (IQR) or n (%)**
Duration since diagnosis of HIV (Months)	360	107.0 (71.5, 132.0)
Range (Min. – Max.)		1.0–181.0
Duration before ART initiation (Months)	360	44.0 (4.5, 78.5)
Range (Min. – Max.)		0.0–166.0
Duration of ART use (Months)	360	88.0 (51.0, 122.5)
Range (Min. – Max.)		1.0–141.0
Duration of current ART (Months)	360	51.0 (17.0, 76.0)
Range (Min. – Max.)		0.0–147.0
ART Line		
First line (NRTI + NNRTI)	360	286 (79.4%)
Second line (NRTI + PI)		74 (20.6%)
Others		
Dapsone		5 (1.4%)
Septrin	359	353 (98.3%)
Septrin/Isoniazid		1 (0.3%)
Suppressed viral load (< 1000 copies/ml)	358	303 (84.6%)
Baseline CD4 cell count per mm^3^	304	243.0 (10.8.0, 399.0)
Range (Min. – Max.)		1.0–1459.0
< 200.0		122 (40.1%)
200.0–499.0	304	132 (43.4%)
≥ 500.0		50 (16.4%)
Current CD4 cell count per mm^3^	360	491.0 (336.5, 701.0)
Range (Min. – Max.)		1.0–1845.0
< 200.0		33 (9.2%)
200.0–499.0	360	153 (42.5%)
≥ 500.0		174 (48.3%)
WHO Clinical stage		
Stage 1		122 (35.6%)
Stage 2	343	61 (17.8%)
Stage 3		132 (38.5%)
Stage 4		28 (8.2%)

*IQR* Inter Quartile Range

N is less than 360 in other variables due to missing data

### Prevalence of HIV-associated neurocognitive disorder

Based on MoCA 292 (81.1%) of the participants had mild HAND, and 68 (18.9%) were normal. Using IHDS, 9 (2.5%) of the participants had severe HAND. There were 13 (3.6%) who had functional impairment.

Diagnosis of HAND using the operational definitions demonstrated that 9 (2.5%) had severe HAND, 283 (78.6%) had mild HAND, and 68 (18.9%) were normal (Table 4).

**Table 4:** Montreal cognitive assessment (MoCA), International HIV dementia scale (IHDS), and the Lawton Instrumental Activities of Daily Living Scale (IADL)

**Item**	**N**	**Mean (SD) or n (%)**
MoCA	360	21.2 (4.2)
Range (Min. – Max.)		12.0–30.0
Mild ANI/MND (MoCA < 26)	360	292 (81.1%)
Normal (MoCA ≥26)		68 (18.9%)
IHDS	360	9.8 (1.7)
Range (Min. – Max.)		5.0–12.0
HAD (IHDS < 10)		9 (2.5%)
No HAD (IHDS ≥10)	360	351 (97.5%)
IADL	360	8.0 (0.3)
Range (Min. – Max.)		5.0–8.0
Experienced functional impairment	360	13 (3.6%)
Activity		
Food preparation/Housekeeping/Laundry		1 (7.7%)
Food preparation/Laundry	13	2 (15.4%)
Responsibility for own medication		6 (46.2%)
Laundry		3 (23.1%)
Shopping		1 (7.7%)
CPE score	360	7.4 (1.8)
Range (Min. – Max.)		5.0–10.0

*SD* Standard Deviation

The participants diagnosed with HAND performed consistently and significantly worse than the cognitively normal group across all domains, except for the domain of orientation (Table 5).

**Table 5:** Comparison of Montreal cognitive assessment (MoCA), and International HIV dementia scale (IHDS) cognitive domains by presence or absence of HAND

**Domains**	**N**	**HAND (*N*** **= 292)**	**No HAND (*N*** **= 68)**	***P*-value**
		**Mean (SD)**		
MoCA				
Executive		2.0 (1.8)	4.3 (1.0)	< 0.001
Naming		2.7 (0.5)	3.0 (0.0)	< 0.001
Attention		4.0 (1.6)	5.8 (0.5)	< 0.001
Language		1.1 (0.6)	1.8 (0.7)	< 0.001
Abstraction		1.0 (0.6)	1.5 (0.6)	< 0.001
Memory		2.4 (1.8)	4.3 (1.0)	< 0.001
Orientation		5.9 (0.2)	6.0 (0.2)	0.415
Total		19.8 (3.4)	27.0 (1.0)	< 0.001
IHDS				
Motor speed		3.6 (0.6)	4.0 (0.2)	< 0.001
Psychomotor speed		3.0 (0.7)	3.7 (0.5)	< 0.001
Memory-recall		2.8 (1.1)	3.8 (0.6)	< 0.001
Total		9.4 (1.6)	11.5 (0.9)	< 0.001

*IHDS* International HIV Dementia, *MoCA* Montreal Cognitive Assessment

### Association between socio-demographic characteristics and diagnosis of HAND

Participants who were diagnosed with HAND were significantly older than those without HAND; 41.9 (SD: 10.6) vs. 33.0 (SD: 12.5) years, *p* < 0.001. This demonstrates a 7% increased chance/risk of diagnosis of HAND among the older participants compared to the younger; OR: 1.07 (95% CI: 1.05, 1.10).

Significantly lower proportion of male participants, and significantly higher proportion of the married participants were diagnosed with HAND compared to those without HAND; 32.5% vs. 45.6%, *p* = 0.042, and 47.6% vs. 33.8%, *p* = 0.040 respectively. These findings show that there was a 42% reduced odds of diagnosis of HAND among the male participants compared to the female, OR: 0.58 (95% CI: 0.34, 0.98) and a 78% increased odds of diagnosis of HAND among the married participants compared to the single/separated/widowed/divorced, OR: 1.78 (95% CI: 1.02, 3.09).

Compared to the participants without HAND, a significantly lower proportion of participants with secondary and tertiary level of education were diagnosed with HAND, 38.7% vs. 61.8%, *p* < 0.001, and 9.9% vs. 25.0%, *p* = 0.001 respectively. Compared to those with primary level of education, the participants with secondary and tertiary level of education had 84, and 90% reduced odds of diagnosis of HAND; OR: 0.16 (95% CI: 0.08, 0.35), and 0.10 (95% CI: 0.04, 0.25) respectively (Table 6).

**Table 6:** Association between socio-demographic characteristics and diagnosis of HAND

**Variable**	**Presence of HAND**		***P*-value**	**UOR (95% CI)**
	**Yes (*n*** **= 292, 81.1%)** **Mean (SD) or n (%)**	**No (*n*** **= 68, 18.9%)** **Mean (SD) or n (%)**		
Age (Years)	41.9 (10.6)	33.0 (12.5)	< 0.001	1.07 (1.05, 1.10)
Male vs. Female	95 (32.5%)	31 (45.6%)	0.042	0.58 (0.34, 0.98)
Married vs. Single/Widowed/separated/divorced	138 (47.6%)	23 (33.8%)	0.040	1.78 (1.02, 3.09)
Education				
Primary	150 (51.4%)	9 (13.2%)	< 0.001	Reference
Secondary	113 (38.7%)	42 (61.8%)	0.001	0.16 (0.08, 0.35)
College	29 (9.9%)	17 (25.0%)	0.001	0.10 (0.04, 0.25)
Occupation				
Unemployed	153 (52.6%)	35 (51.5%)	0.869	Reference
Self employed	71 (24.4%)	18 (26.5%)	0.722	0.90 (0.48, 1.70)
Employed	67 (23.0%)	15 (22.1%)	0.864	1.02 (0.52, 2.00)
Income (Ksh.per Month)				
> 10,000	39 (13.6%)	8 (12.7%)		Reference
≤ 10,000	247 (86.4%)	55 (87.3%)	0.843	0.92 (0.41, 2.08)

*UOR* Unadjusted Odds Ratio, *95% CI* 95% Confidence Interval

### Association between clinical characteristics and diagnosis of HAND

There was no evidence of a difference in BMI, baseline CD4 levels, current CD4 levels, WHO clinical stage, and presence of comorbidities between those who were diagnosed with HAND and those who did not have HAND, *p* > 0.05.

The proportion of participants with suppressed viral load were similar for those who were diagnosed with HAND, and those who were normal, 85.5% vs. 80.9%, *p* = 0.340 respectively.

The average CPE score was significantly higher among those who were diagnosed with HAND compared to those without HAND; 7.6 (SD: 1.8) vs. 6.8 (SD: 1.7), *p* < 0.001. The crude estimates show that the participants who had higher CPE score were associated with 30% increased odds of being diagnosed with HAND; OR: 1.30 (95% CI: 1.11, 1.53) (Table 7).

**Table 7:** Association between clinical characteristics and diagnosis of HAND

**Variable**	**Presence of HAND**		***P*-value**	**UOR (95% CI)**
	**Yes (*n*** **= 292, 81.1%)** **Mean (SD) or n (%)**	**No (*n*** **= 68, 18.9%)** **Mean (SD) or n (%)**		
BMI (Kg/m^2^)				
< 18.5	24 (8.2%)	10 (14.7%)	0.099	0.56 (0.25, 1.27)
18.5–25.0	179 (61.3%)	42 (61.8%)	0.944	Reference
25.0–30.0	73 (25.0%)	13 (19.1%)	0.306	1.32 (0.67, 2.60)
> 30.0	16 (5.5%)	3 (4.4%)	0.723	1.25 (0.35, 4.49)
Baseline CD4 (cells per mm^3^)				
< 200	103 (42.0%)	19 (32.2%)	0.166	Reference
200–500	105 (42.9%)	27 (45.8%)	0.686	0.72 (0.38, 1.37)
> 500	37 (15.1%)	13 (22.0%)	0.197	0.53 (0.24, 1.17)
Current CD4 (cells per mm^3^)				
< 200	27 (9.3%)	6 (8.8%)	0.913	Reference
200–500	121 (41.4%)	32 (47.1%)	0.398	0.84 (0.32, 2.21)
> 500	144 (49.3%)	30 (44.1%)	0.440	1.07 (0.41, 2.81)
WHO Clinical stage				
Stage 1	95 (34.4%)	27 (40.3%)	0.367	Reference
Stage 2	47 (17.0%)	14 (20.9%)	0.458	0.95 (0.46, 1.99)
Stage 3	110 (39.9%)	22 (32.8%)	0.289	1.42 (0.76, 2.66)
Stage 4	24 (8.7%)	4 (6.0%)	0.465	1.71 (0.54, 5.34)
Have comorbidities	23 (7.9%)	5 (7.4%)	0.879	1.08 (0.40, 2.96)
Regimen				
First line (NRTI + NNRTI)	241 (82.5%)	45 (66.2%)		Reference
Second line (NRTI + PI)	51 (17.5%)	23 (33.8%)	0.003	0.41 (0.23, 0.74)
Years on current HAART	4.4 (3.0)	3.2 (2.8)	0.002	1.16 (1.05, 1.28)
Individual HAART drugs				
EFV/ETR/ABC	139 (47.6%)	35 (51.5%)	0.565	Reference
AZT	127 (43.5%)	18 (26.5%)	0.010	2.99 (0.49, 18.01)
TDF	161 (55.1%)	48 (70.6%)	0.020	1.93 (0.33, 11.15)
NVP	108 (37.0%)	12 (17.7%)	0.002	2.28 (1.11, 4.67)
Years since HIV diagnosis	8.1 (3.7)	8.4 (3.7)	0.620	0.98 (0.91, 1.06)
Viral load < 1000 copies/ml	248 (85.5%)	55 (80.9%)	0.340	1.40 (0.70, 2.77)
CPE score	7.6 (1.8)	6.8 (1.7)	< 0.001	1.30 (1.11, 1.53)

*UOR* Unadjusted Odds Ratio, *95% CI* 95% Confidence Interval

### Logistic regression model assessing the determinants of diagnosis of HAND

The level of income was retained in the model although it was not statistically significant. This is because income level confounded the effect of gender.

Years of current HAART, use of second line regimen, viral load suppression (< 1000 copies/ml) and CPE score were retained despite them being not statistically significant since they clinically meaningful variables that are known to affect HAND.

The findings demonstrate that older participants were associated with 6% increased odds of diagnosis of HAND compared to the younger participants, OR: 1.06 (95% CI: 1.03, 1.10) and male participants were associated with 52% reduced odds of being diagnosed with HAND, OR: 0.48 (95% CI: 0.24, 0.97).

Education level was associated with diagnosis of HAND. The findings show that participants who had secondary level of education and those who had tertiary level of education were associated with 84 and 89% reduced odds of being diagnosed with HAND, OR: 0.16 (95% CI: 0.07, 0.38) and 0.11 (95% CI: 0.04, 0.35) respectively.

Compared to low WHO clinical stage (stage 1 or 2), the advanced WHO clinical stage (stage 3 or 4) was associated with more than twice increased odds of being diagnosed with HAND, OR: 2.45 (95% CI: 1.20, 5.01).

After adjusting for age, gender, education level, income, years on the current HAART, use of second line regimen, WHO clinical stage, and viral load level, the effect of CPE score was removed, AOR: 1.13 (95% CI: 0.89, 1.42) (Table 8).

**Table 8:** Logistic regression model assessing the determinants of diagnosis of HAND

**Variable**	**Unadjusted Estimates**		**Adjusted Estimates**	
	**OR (95% CI)**	***p*-value**	**OR (95% CI)**	***p*-value**
Age (Years)	1.07 (1.05, 1.10)	< 0.001	**1.06 (1.03, 1.10)**	**< 0.001**
Male	0.58 (0.34, 0.98)	0.042	**0.48 (0.24, 0.97)**	**< 0.001**
Education level				
Secondary vs. primary	0.16 (0.08, 0.35)	0.001	**0.16 (0.07 0.38)**	**< 0.001**
Tertiary vs. Primary	0.10 (0.04, 0.25)	0.001	**0.11 (0.04, 0.35)**	**< 0.001**
Income ≤ Ksh/Month10000	0.92 (0.41, 2.08)	0.843	0.54 (0.19, 1.54)	0.252
Years on current HAART	1.16 (1.05, 1.28)	0.002	1.04 (0.90, 1.19)	0.627
On Second line regimen	0.41 (0.23, 0.74)	0.003	0.66 (0.30, 1.43)	0.288
WHO stage 3/4 vs. 1/2	1.49 (0.86, 2.57)	0.152	**2.45 (1.20, 5.01)**	**0.014**
Viral load < 1000 copies/ml	1.40 (0.70, 2.77)	0.340	0.70 (0.28, 1.73)	0.439
CPE score	1.30 (1.11, 1.53)	< 0.001	1.13 (0.89, 1.42)	0.312
**Sample Size**			**331**	

*OR* Odds Ratio, *CI* Confidence Interval

## Discussion

The prevalence of HAND in the study was 81.1%. Mild HAND was 78.6% and severe HAND 2.5%. The current literature depicts rising prevalence of the milder forms of HAND and decrease in severe form of HAND [16–18].

The screening tools for HIV-associated neurocognitive disorders among adults living with HIV in sub-Saharan Africa perform well in screening for severe forms of HAND, but lack sensitivity and specificity for mild forms of HAND [19].

The overall prevalence is also higher compared to published literature but so far MoCA is the best tool for assessing mild neurocognitive impairment in the absence of new validated HAND screening tools in Sub-Saharan Africa. The ‘gold standard’ neuropsychological test battery is burdensome and rarely available in many African countries. To address this gap, many brief cognitive assessments have been developed and can be administered by trained personnel to identify the suspected cases among the high-risk population cohort for further formal diagnostic procedures.

Different viral clades may also account for the variation in HAND as certain clades maybe more or less neuropathogenic [17, 20, 21]. Neurocognitive deficit is more prevalent in regions where subtype C HIV predominates and this subtype is predominant in Sub- Saharan Africa. However, the viral clades were not studied in the present study.

Socio-demographic characteristics are important health features that affect the prevalence of HAND. Various studies on HAND in Africa included a relatively similar population with a mean age ranging from 29.75 to 40 years [22].

Older age was associated with HAND. This was similar to a study done in Zimbabwe [23], Thailand [24], and China [25]. This is thought to be due to the neurocognitive decline that comes with aging.

Men were less likely to have HAND. This was comparable to studies in Zambia [26] and Nigeria [27]. This is because of genetic and social factors. In the pathogenesis of HAND men have less immune activation of the macrophages, astrocytes and microglia hence less toxin signaling pathways that underlie the brain dysfunction in HAND. Most men in our African society are privileged to go to school and get educated while female play a role of doing house chores hence they have better cognitive reserve than women hence better cognitive performance [28].

Majority of the participants were married, unemployed with low level of income and this represents a low socio-economic status of the patients. This was a predictor of poor neurocognitive performance as seen in a study in Cameroon [29].

Higher level of education was associated with less HAND. This was in line with findings from a systematic review in sub Saharan Africa [30], United States of America [16], and in South Africa [31]. Participants with higher level of education have better scoring in the screening tests, better awareness about the chronicity of HIV and good follow up resulting in good ART adherence and a reduced risk of HAND.

Assessment of the stage of HIV infection with WHO clinical staging, CD4 count and viral load is an important element in the evaluation of HAND.

Majority of the participants were in WHO clinical stages 3 and 4. Advanced WHO clinical stage 3 or 4 was associated with more than twice increased odds of being diagnosed with HAND. This was similar to other studies, which showed increased rate of HAND with advanced stages of HIV infection in Nigeria [32, 33], and in Uganda [34].

There is evidence of advanced immunosuppression leading to a higher incidence of HIV associated brain injury and also most patients present to the hospitals with late stage of HIV infection and advanced neurocognitive impairment.

Studies in the HAART era have shown that current CD4 counts have no correlation and are not predictive of HAND [35]. Current CD4 count was significant in the Pre HAART era. In the above study it did not appear as an important marker.

The baseline CD4 cell count informs the likelihood that progressive cognitive impairment is due to HAD, which occurs at counts of < 200 cells per mm^3^ in untreated patients. From the study, the Prevalence of HAD was very low and could explain the lack of association.

The proportion of participants with suppressed viral loads < 1000 copies per ml were similar in both those with HAND and the normal participants. The findings were similar to others, which revealed that markers such as plasma viral load are not associated with HAND [4, 35–37].

This shows that normalization of immune indices that reflect peripheral immune function does not adequately reflect the environment that continues to exist in the CNS. CSF viral load has shown promise as a predictor of HAND but more studies need to establish the association.

In the study, CPE score was not associated with HAND after adjusting for the other variables. This was similar to findings by Marra et al. and Cysique et al. [38, 39].

The cross sectional study design limited meaningful interpretation of the relationship between CPE scores and the presence of HAND since the timing of treatment initiation and duration of treatment need to be considered. Besides, the national ART treatment guidelines determine the initial ART regimen choice and thereby the CPE scores.

### Study limitations

Neuropsychological test battery is the gold standard for diagnosis of HAND; however the availability of these tests together with the expertise needed for their administration is limited in our set up. We therefore chose to use screening tools that have been validated locally (using neuropsychological test battery as gold standard) as surrogate diagnostic screening tests. These tests have moderate utility in diagnosing HAND but are easy to use in routine clinical practice and allows for comparison of our findings with previous studies that have used similar tools.

Finally, there are potential confounders, which we did not assess for as baseline such as the nadir CD4 cell count.

## Conclusion and recommendation

The prevalence of HAND remains high in this HAART error with a higher prevalence of mild HAND (78.6%) and low prevalence of severe HAND (2.5%).

The independent factors associated with HAND are age, gender, level of education and WHO clinical stage.

Therefore there is need for early screening for HAND in HIV infected patients and a future prospective study to help understand the true association between HAND and the CPE score of ART regimen.

## Supplementary information

**Additional file 1.** Study Questionnaire –Demographic Data.

## Data Availability

The datasets used during the study are available from the corresponding author on reasonable request.

## Abbreviations

AMPATH: Academic Model Providing Access to Healthcare
ANI: Asymptomatic Neurocognitive Impairment
ABC: Abacavir
ART: Antiretroviral therapy
ARV: Antiretroviral drugs
ATV-r: Atazanavir/ ritonavir
AZT: Zidovudine
BMI: Body Mass Index
CNS: Central Nervous System
CD4: Cluster of differentiation 4
CPE: Central nervous system Penetration Effectiveness
ETR: Etravirine
EFV: Efavirenz
HAD: HIV-Associated Dementia
HAND: HIV-Associated Neurocognitive Disorders
HAART: Highly Active Antiretroviral Therapy
IADL: Lawton Instrumental Activities of Daily Living
IHDS: International HIV Dementia Scale
LPV-r: Lopinavir/ ritonavir
MND: HIV-associated Mild Neurocognitive Disorder
MoCA: Montreal Cognitive Assessment tool
MTRH: Moi Teaching and Referral Hospital
NVP: Nevirapine
NRTI: Nucleoside reverse transcriptase inhibitors
NNRTI: Non-nucleoside reverse transcriptase inhibitors
PI: Protease Inhibitors
SSA: Sub Saharan Africa
TDF: Tenofovir
3TC: Lamivudine
