# Supplementary material for: HIV-associated neurocognitive disorders at Moi teaching and referral hospital, Eldoret, Kenya
Source: BMC Neurol. 2020 Jul 14;20:280. doi: 10.1186/s12883-020-01857-3 (PMC7359564; doi:10.1186/s12883-020-01857-3)
Supplement: Supplementary file 1 — Additional file 1. Study Questionnaire –Demographic Data. [file 12883_2020_1857_MOESM1_ESM.docx]

**Additional file 1**

## Appendix 1: Study Questionnaire –Demographic Data

1. PID AMRS HT (cm) WT(kg) BMI
2. GENDER MALE FEMALE
3. DOB (DMY) ........../............/................ AGE -------------------
4. EDUCATION LEVEL ACHIEVED Primary

Secondary

Tertiary

**Specify level (University-(Diploma,Bachelors,Masters,PHd),College, Technical Institute)-------------**

1. WHO Clinical Staging I

II

III

IV

1. BASELINE CD4 DATE CURRENT CD4 DATE

COUNT ( DMY) COUNT (DMY)

**------------- ------------- ------------- -------------**

1. CURRENT VIRAL LOAD DATE

COPIES/ML (DMY)

­­­­­­­­­----------------- **-------------**

8. DRUG REGIMEN 1^ST^LINE 2^ND^ LINE 3^RD^ LINE

________________________________ ________________

Other-Specify______________________________________________________

9. Which date of first diagnosis of HIV---------------------- Duration of HIV infection ...............................................

10. Year of initiation of ART ___________Duration of HAART______________

Current ARV regimen start date ________________ Duration__________

1. Marital Status:

Married

Widowed

Divorced/Separated

Single

1. Do you live alone

YES

NO

(If NO please specify------------)

1. Employment Status Employed

Self Employed

Unemployed

1. Level of Income(KSHS per month)

0 to 10,000

10,000 to 50,000

50,000 to 100,000

Above 100,000

15 .Have you Suffered from Any Infectious illness in the Last 1 year and got admitted?If Yes,please specify the illness and whether you are still on medication.

YES ­­­­­­­------------------

NO

16 .Do you suffer from any other chronic illness for which you take medication such as High Blood Pressure,Diabetes, Mental illness?If Yes,please specify,with duration of taking those drugs

YES -------------------

NO

17.Do you consume Alcohol or any Substance of Abuse such as Bhang,Cocaine,Heroine?If Yes,please specify

YES -----------------

NO

18.When did you last take alcohol? ------------- How much of alcohol do you take per sitting?------------- CAGE score--------

19.Have you ever suffered injury on the head with loss of consciousness for more than 30 minutes?

YES

NO
